# SHOX Duplication and Tall Stature in a Patient with Xq Deletion and Vascular Disease

**DOI:** 10.1155/2019/2691820

**Published:** 2019-04-08

**Authors:** J. M. Ramirez, F. A. Rodríguez, M. I. Echeverría, A. L. Vargas, A. E. Calderón, R. M. Miatello, N. F. Renna

**Affiliations:** ^1^Medical Genetic Institute, School of Medicine, University of Cuyo, Argentina; ^2^Maternal and Child Research Institute, School of Medicine, University of Chile, Santiago, Chile; ^3^Pathophysiology Area, Pathology Department, School of Medicine, National University of Cuyo, Mendoza, Argentina; ^4^IMBECU-CONICET, Argentina; ^5^Coronary Unit, Cardiology Center, Spanish Hospital from Mendoza, Mendoza, Argentina

## Abstract

The anomalies of X chromosome are classified as numerical or structural. Concomitant structural anomalies in this chromosome that associate partial loss of its long arm with duplications in its short arm are uncommon. Only a few cases have been published and in most of them the reported patients present ovarian dysfunction, tall stature, and overdosage of the SHOX gene with locus Xp22.33. Considering these reports, we evaluated the case of a woman with a deletion in the long arm of the X chromosome, premature ovarian failure, tall stature, and multiple arterial vascular disease. With the aim to find a relationship between karyotype and phenotype, we explored associated anomalies in Xp and certified the overdosage of the SHOX gene in this case by MLPA. Also, taking into account the fact that the gene locus of the angiotensin-converting enzyme type 2 (ACE2) is located in Xp, our goal was to investigate the influence of this gene in the development of cardiovascular disease. The detection of the gene product of ACE2 by ELISA was undetectable. We have proposed that cytogenetic anomalies in X chromosome could contribute to decrease this protein synthesis in this gender.

## 1. Introduction

The anomalies of X chromosome are classified as numerical or structural and can affect short arm (Xp) or long arm (Xq). The most studied are cluster under the name of Turner syndrome (TS). This group includes monosomy as the majority but also losses (deletions), duplications, inversions, and chromosomal rings that affect the X chromosome. The increase in morbidity and mortality is a cardinal feature in patients with TS, describing in them a risk of premature death three times greater than that of the general population and life expectancy decreased by at least a decade [1]. Patients with TS have multisystemic complications [2] but cardiovascular disease is the main cause of the decline in life expectancy [1].

The correlation between karyotype and phenotype expression in this group of patients has been probed [3]. Concomitant structural abnormalities in X chromosome that include partial Xq deletion with Xp duplication are uncommon. In the few cases reported, ovarian dysfunction associated with very tall stature has been frequent clinical features [4–6]. In the majority of these patients, an overdosage of SHOX gene associated with the condition was certified. In the evaluation of these cases, some authors have reported that an extra copy of SHOX gene could be necessary but not enough for the development of tall stature in similar patient with gonadal dysgenesis [7]. The SHOX gene (short stature homeobox-containing gene) is most strongly expressed in marrow fibroblast but is widely expressed in limbs, pharyngeal arches, and osteogenic cells too and is implied in the final height determination [8, 9]. SHOX is located on pseudoautosomal region (PAR1) in sexual chromosomes (Xp22.3 and Yp11.3). Genes located in PAR1 are represented by two active copies producing a dosage effect of the SHOX gene in patients with sexual chromosomes aberrations. This is due to the fact that several loci in Xp22.3 suffer an incomplete inactivation [10]. Depending on the altered SHOX dosage, haploinsufficiency causes short stature while overdosage contributes to tall stature [11].

The precise mechanisms of increased cardiovascular risk in TS remain unclear [12]. Different authors have proposed several candidates genes as TGF*β*, VEGF, etc. but their participation in the pathophysiology of disease is unknown [13, 14].

Considering that the locus of the angiotensin I converting enzyme type 2 (ACE2) is located in the short arm of the X chromosome, we proposed to investigate the influence of this gene in the development of endothelial dysfunction and cardiovascular disease in a patient who consulted by premature ovarian failure, tall stature, and multiple and severe arterial vascular disease for her age. The influence of ACE2 gene has not been studied in patients with X chromosomes aberrations. Also, considering that, in similar cases reported, deletions in Xq could be associated with duplications of the SHOX gene in Xp; we certified that tall stature in our patient is associated with duplication of this gene through MLPA (multiplex ligation- dependent probe amplification) methodology.

## 2. Case Report

The proband is 28 years old and is the only daughter of nonconsanguineous parents. A maternal height of 152 cm and a paternal height of 161 cm were recorded. Among her antecedents she presents secondary amenorrhea, primary hypothyroidism, hyperinsulinism, insulin resistance, and premature ovarian failure. The complementary tests showed uterine hypoplasia and small ovaries and hypergonadotrophic hypogonadism, with normal androgenic profile and normal growth hormone (GH). Her serum luteinizing hormone (LH) and follicle-stimulating hormone (FSH) were 23.20 mIU/ml and 23.90 mIU/ml, respectively. Her serum estradiol was <10mg/ml. Her serum testosterone and dehydroepiandrosterone were normal values (0.28 ng/ml and 107 ug/dl, respectively). Her serum insulin-like growth factor-1 (IGF-1) was normal (327 ng/ml).

**Figure 1:**
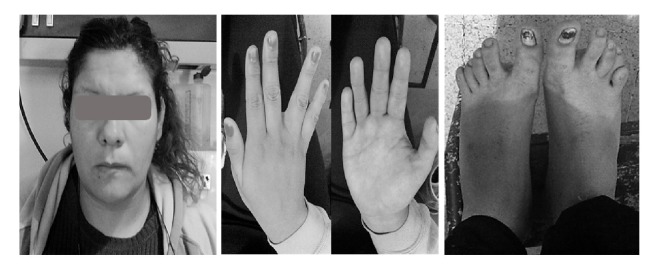
*Physical examination:* note frontomalar prominence in the face. In the upper limbs there was cutaneous syndactyly of the lower third and bilateral fifth finger clinodactyly. In lower limbs there was separation of the first toe; brachydactyly of the 4th and 5th toes in the left foot and in the right foot 5th finger was overlapping over the 4th.

The karyotype in peripheral blood lymphocytes showed 46, X,del(X),(q22-q27). In the physical examination she presented macrocephaly, upslanting palpebral fissures, and frontomalar prominence. Acanthosis nigricans were observed in the neck. In the upper limbs there was cutaneous syndactyly of the lower third and bilateral fifth finger clinodactyly. In lower limbs there was separation of the first toe; brachydactyly of the 4th and 5th toes in the left foot and in the right foot 5th finger was overlapping over the 4th (Figure 1). Her height was 186 cm and her weight was 97 kg (weight and height were above the 97th centile).

## 3. Methods

### 3.1. Vascular Ultrasound

A carotid ultrasound scan was performed with a Siemens Acuson X700 ultrasound machine equipped with vascular health software (syngo arterial health package) and linear transducer from 7.5 to 12MHz. The artery is scanned and the diameter is measured, in the longitudinal section, in three regions (CCA: common carotid artery, CB: carotid bulb, and ICA: internal carotid artery). B-mode ultrasound images of the CCA, CB, and ICA segments were obtained and the image files were recorded. To determine the parameters of dynamic endothelial dysfunction in the brachial artery (FMD: flow-mediated dilatation) the nondominant arm was placed in the patient in a permanent and immobilized position to allow access to the brachial artery with the transducer. The artery image was obtained 3 to 7 cm above the antecubital fold with a linear transducer with a frequency of 7.5 MHz. The measurements are coordinated at the end of diastole. After the determination of the arterial diameter at basal, a pressure cuff was placed distal to the transducer and applied at a pressure of 200 mm Hg for 5 min and then quickly released the occlusion. At the first minute of release of the occlusion, the arterial diameter was measured to evaluate the response to hyperemia.

### 3.2. SHOX Gene Analysis by MLPA: PARs Analysis

The Multiplex Ligation-dependent Probe Amplification (MLPA) probemix P018-G1 SHOX was employed to search for PAR1/PAR2 deletions and duplications, using conditions specified by the manufacturer (MRC-Holland, Amsterdam, The Netherlands; http://www.mrc-holland.com). This kit contains probes for each exon of* SHOX*, as well as probes upstream and downstream of* SHOX*, where* SHOX* regulatory elements are located (CNE). Furthermore, several probes in the X-specific region of the X chromosome were included to characterize large deletions. Finally, nine autosomal reference probes were included for normalization. The MLPA data analysis was performed using Coffalyser. Net software is freely downloadable at www.mlpa.com. A value below 0.7 or above 1.3 is regarded as indicative of a heterozygous deletion (copy number change from two to one allele) or duplication (copy number change from two to three or more alleles), respectively.

### 3.3. Measure of ACE2 by ELISA

We used an enzyme-linked immunosorbent assay (ELISA) for quantitative determination of ACE2 in human urine samples provided for BioSource. Polyclonal antibody specific for human ACE2 has been precoated onto 96-well microplate. Standards and sample are pipeted into the wells and any ACE2 present is bound by immobilized antibody. Bound ACE2 is captured by biotinylated anti-human ACE2 polyclonal antibody. HRP conjugated streptavidin is added. After washing, a substrate solution is added. The colors develop in proportion to the bounded ACE quantity. The color development is stopped and the intensity of color is measured.

## 4. Results

**Figure 2:**
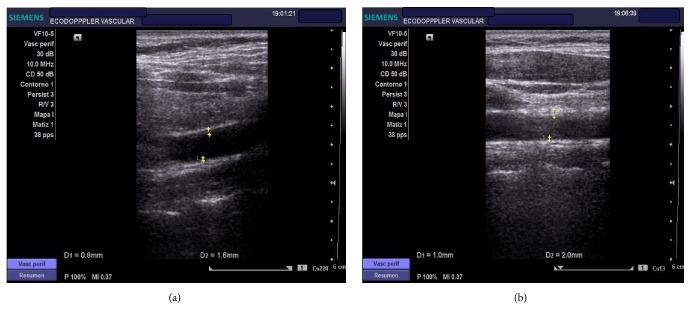
The vascular ultrasound study in our patient showed atherogenic plates: (a) 1.6 mm in RCCA (right common carotid artery) and (b) 2 mm in LCCA (left common carotid artery).

**Figure 3:**
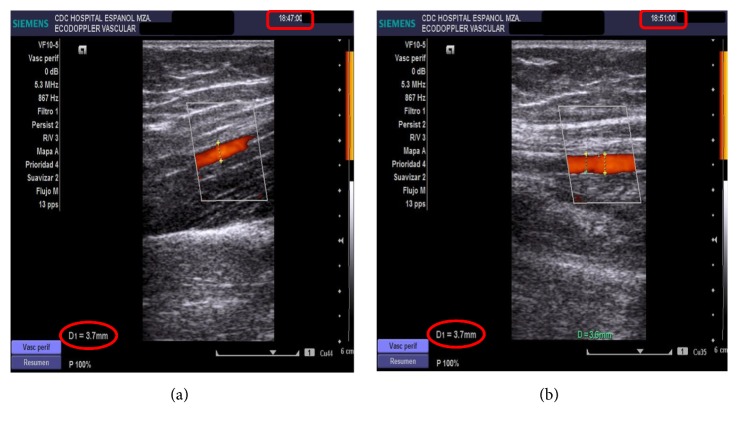
In FMD (flow-mediated dilation) in brachial artery patient did not present vascular reactivity in the first minute and this was interpreted as endothelial dysfunction.

The vascular ultrasound study in our patient showed an atherogenic plate of 1.6 mm in RCCA (right common carotid artery) and 2 mm in LCCA (left common carotid artery) (Figure 2). In FMD in brachial artery our patient did not present vascular reactivity in the first minute and this was interpreted as endothelial dysfunction (Figure 3).

**Figure 4:**
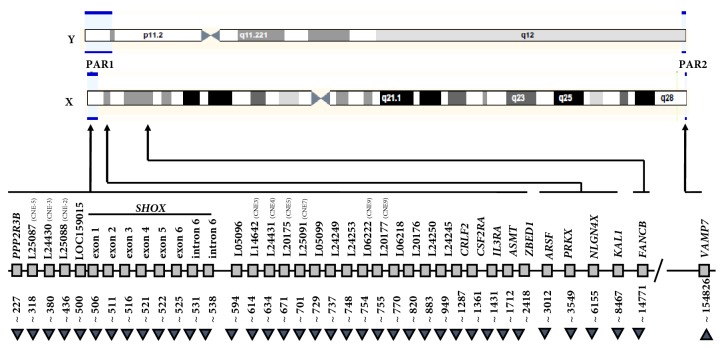
*PARs alterations detected by MLPA.* (Up) PARs are depicted with blue lines over sexual chromosome diagram; (middle) name and position (Kb from p-telomere) of each MLPA SALSA P018-G1 probe are indicated with a grey square (diagram not drawn to scale); (down) MLPA analysis result where black triangle (▲) reduced copy number (<2) and inverted black triangle (▼) increased copy number (>2) of the MLPA specific probe.* CNE: conserved noncoding elements.*

**Figure 5:**
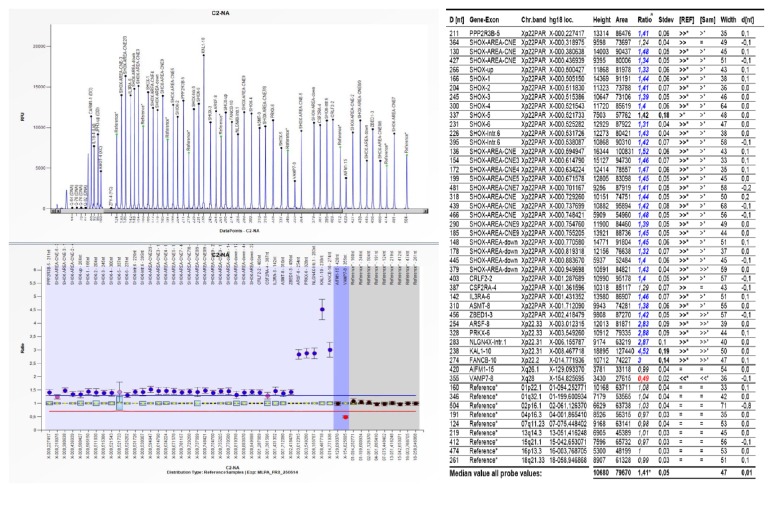
Multiple ligation-dependent probe amplification (MLPA) analysis was performed using a SALSA MLPA P018-G1. Reference Kit (MRC-Holland, Amsterdam, The Netherlands) according to the manufacturer's instruction. The MLPA data analysis was performed using Coffalyser. To right Coffalyser sheet showing the ratios calculated for each probe. A value below 0.7 or above 1.3 is regarded as indicative of a heterozygous deletion (copy number change from two to one allele) or duplication (copy number change from two to three or more alleles), respectively. This kit contains probes for each exon of* SHOX*, as well as probes upstream and downstream of* SHOX*, where* SHOX* regulatory elements are located (CNE). Furthermore, several probes in the X-specific region of the X chromosome were included to characterize large deletions. Finally, nine autosomal reference probes were included for normalization.

**Figure 6:**
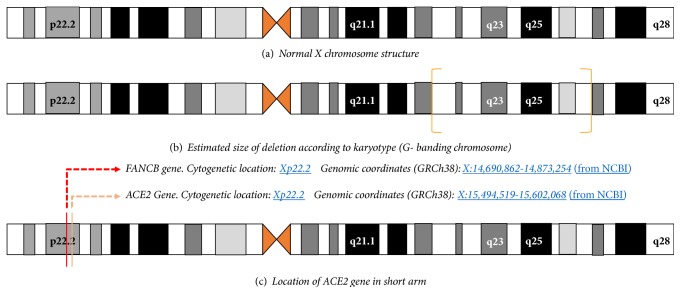
(a) X chromosome diagram. (b) In orange brackets, size of deletion in long arm of X chromosome is delimited. No aberrations in short arm were informed. (c) The location of different genes (FANCB and ACE2) is marked in diagram of X chromosome (short arm). Duplication informed by MLPA involves the entire PAR1 and spread until FANCB gene (red dashed arrow). ACE2 gene (blue dashed arrow) is located next to FANCB. The activity of ACE2 was measured by ELISA methodology.

MLPA analysis confirmed the duplication that involve the entire PAR1 region and spread to the FANCB gene located in Xp22.2 (Figures 4 and 5). Note the extension of the duplicated region; FANCB gene locus is really close to ACE2 gene, but the latter has a more centromeric position (Figure 6). It is necessary to emphasize that the MLPA Kit does not have control sequences for the ACE2 gene; for this reason the ELISA technique was used, in order to indirectly determine alterations in gene function. For another hand a haploinsufficiency of VAMP7 gene was detected in long arm of X chromosome (Figures 4 and 5). This allowed us to specify that the size of deletion in the long arm of the X chromosome spread to a more terminal position than that defined by G banded karyotype.

The value of ACE2 in urine by ELISA was undetectable. The measurement of ACE2 was compared with the standard value published by the manufacturer for normal controls (ACE2 levels range in urine in healthy donors from 1 to 10 ng/ml). Female and male controls evaluated in our group demonstrated values contained in the normal range, including the parents of the patient (median of 4 pg/ml). In addition, the value of the sample was compared with women with secondary amenorrhea who had normal karyotype (who showed lower values but in normal range of ACE2) and men with Klinefelter syndrome with karyotype 47, XXY (who presented values higher than the normal range of ACE2).

## 5. Discussion

SHOX gene is known to play an important role in grown determination in human beings. The clinical signs as short stature, gonadal dysgenesis, and somatic malformations in patients with Turner syndrome are caused by partial or total haploinsufficiency of one of the X chromosomes. Ogata et al. [5] concluded that the combination between SHOX overdosage and estrogen deficiency was necessary to explain tall stature in many patients. According to del Rey G et al. [9], the combination of SHOX gene triplication and estrogen deficiency is not always produce tall stature. The heterogeneity in phenotype of different patients could be due to certain variants in their karyotype and we should consider the role of X-inactivation center where genes located near the breakpoints are also affected. In our patient, considering the cytogenetic formula and adding her clinical features, the SHOX gene overdosage probably explains her tall stature in a context of estrogen deficiency.

Among the candidate genes, those involved in the renin angiotensin system (RAS) have been some of the most studied in different attempts to establish their participation in pathophysiology of cardiovascular disease. However, its influence on the vascular disease of patients with X chromosome aberrations is not yet clear. We present the case of a patient with Turner syndrome where the aim was to analyze the relationship between karyotype and cardiovascular phenotype. We evaluate cardiovascular risk using vascular ultrasound Doppler and dosage of ACE2 by ELISA technique considering that the locus of ACE2 gene is located in short arm of X chromosome. The measurement of his protein by ELISA was undetectable in this patient. Cardiovascular studies have shown that a rapid and nonnuclear signaling pathway is involved in the estrogen-mediated protection against diseases. This signaling has been studied in vascular and endothelial smooth muscle cells where the coupling between estrogen and its receptor causes the rapid activation of endothelial nitric oxide synthase (eNOS) and production of nitric oxide (NO) [15, 16]. For another hand, experimental animal and clinical researches suggest that the components of RAS are markedly affected by gender [17, 18]. Evidence demonstrated that premenopausal women, as compared to aged-matched men, are protected from cardiovascular disease [19]. With growing age, this cardiovascular protection is lost [20]. This has been demonstrated in the work of Bukowska et al. [20], where the administration of 17*β*-estradiol in human atrial myocardiocytes culture substantially modulates the local RAS via, increases the amount of mRNA ACE2, and simultaneously causes upregulation in the expression of AT2R (angiotensin receptor type 2) and MAS receptor. Particularly in our case, hypoestrogenism could contribute to the decrease ACE2 values in urine, considering that, according to the literature, the concentration of ACE2 protein is proportional to the amount of estrogen in women. However, the undetectable values of ACE2 in our patient allow us to suppose that the type of cytogenetic anomaly probably contributes to decreasing the protein synthesis, especially when compared with women with amenorrhea and normal karyotype who present normal values of ACE2 protein. The hypoestrogenism could explain also vascular disease and endothelial dysfunction. Our results obtained in the vascular ultrasound in the brachial artery are coincident with those published in the literature considering that the absence of flow-mediated dilation (vascular reactivity) is a mechanism dependent of NO.

The literature cites that deletions in long arm of X chromosome (Xq) can manifest varied alterations in the cycles (menstrual irregularities, amenorrhea, or premature ovarian failure). It has also been described that the loss of segment Xq26 can destabilize the short arm and there are few cases of descriptions with deletion in this region associated with duplication in the short arm (Xp). The duplication of genes in short arm for our patient is nearby to ACE2 gene locus. The G banded karyotype's resolution did not allow identifying this kind of alterations that were clinically suspected in our case. The duplication of the SOHX gene, as well as other genes located in Xp, was certified trough MLPA methodology. This same technique also allowed specifying the size of the deletion in Xq through control sequences located in the long arm of the chromosome.

In conclusion, we emphasize the importance to assess the clinical characteristics and phenotype of the patient when conducting different genetic studies. Finally, we support that, in patients with similar clinical features and deletions in Xq, the need to rule out associated alterations in Xp using more precise techniques such as MLPA or array-CGH (array based comparative genomic hybridization) should be considered.
